# Assessment of quality of life in cancer patients using anchoring vignettes: comparisons between mixed cancer patients, patients receiving palliative care, and the general population

**DOI:** 10.3389/fpsyg.2025.1439655

**Published:** 2025-06-05

**Authors:** Andreas Hinz, Ulrich Wedding, Thomas Schulte, Michael Friedrich, Anja Mehnert-Theuerkauf, Astrid Schnabel, Florian Lordick

**Affiliations:** ^1^Department of Medical Psychology and Medical Sociology, Comprehensive Cancer Center Central Germany, University Medical Center Leipzig, Leipzig, Germany; ^2^Department of Palliative Care, Comprehensive Cancer Center Central Germany, Jena University Hospital, Jena, Germany; ^3^Rehabilitation Clinic Bad Oexen, Bad Oeynhausen, Germany; ^4^Department of Medicine (Oncology, Gastroenterology, Hepatology, Pulmonology), Comprehensive Cancer Center Central Germany, University Medical Center Leipzig, Leipzig, Germany

**Keywords:** palliative care, quality of life, self-rated health, response shift, anchoring vignettes

## Abstract

**Objective:**

Quality of life (QoL) has become a relevant outcome criterion in oncology in general and in palliative care in particular. The aims of this study were to compare the QoL of cancer patients receiving palliative care with groups of mixed cancer patients and with the general population, and to test whether response shift effects influence the assessment of QoL.

**Methods:**

This study included data from several cross-sectional investigations: one sample of 152 cancer patients receiving palliative care, two samples of patients with mixed cancer diagnoses (*n* > 500), and two samples of the general population (*n* > 1,000). QoL was assessed with the EORTC QLQ-C30 and with two anchoring vignettes for identifying response shift.

**Results:**

QoL was highest in the general population (EORTC QLQ-C30 mean sum score *M* = 87.4), followed by the mixed cancer patients (*M* = 70.9) and the palliative care group (*M* = 58.2). Both groups of cancer patients rated the anchoring vignette, which presented a subject with mainly physical problems, as being healthier than the general population did.

**Conclusion:**

The results show in which specific dimensions advanced cancer patients report strong detriments in QoL. The different assessments of the vignettes indicate a response shift effect so that the cancer patients have changed their frames of reference for assessing QoL in such a way that they indicate less severe restrictions. This means that the reductions in QoL in cancer patients, as measured with standard questionnaires, tend to underestimate the true detriments.

## Introduction

Quality of life (QoL) has become an important outcome criterion in oncological research and practice (Osoba, 1999). Patients suffering from cancer frequently experience limitations to their QoL as a result of disease and treatment (Bottomley et al., 2005). These limitations in QoL can persist over years (Firkins et al., 2020; Adam et al., 2018). Comparisons between samples of cancer patients and those of the general population show that QoL of the cancer patients is impaired in all of the 15 dimensions of QoL that are covered by the EORTC QLQ-C30 questionnaire (Hinz et al., 2018). Multiple studies have been performed to determine the effects of sociodemographic and clinical factors on QoL in cancer patients (Cihan and Ozturk, 2021; Andreu et al., 2022; Popovic et al., 2013).

It is well-known that cancer patients with advanced, incurable cancer and palliative treatment orientation have particularly severe limitations in their QoL (Beernaert et al., 2016; Vogt et al., 2021; Kyranou and Nicolaou, 2021; Valero-Cantero et al., 2023). Maintaining the highest possible level of QoL in these patients is a central objective of cancer and palliative care (Hoomani Majdabadi et al., 2022). The heterogeneity of detriments in QoL among advanced cancer patients requires early identification and referral of patients with high need of palliative care (Radbruch et al., 2020; Lee et al., 2022).

While there are numerous studies on QoL, systematic comparisons between cancer patients receiving palliative care, other cancer patients, and the general population are rare. Moreover, the sample sizes of studies with cancer patients receiving palliative care are sometimes relatively low, often below *n* = 100, e.g., (Conrad et al., 2017; Estacio et al., 2018; Valero-Cantero et al., 2023; Westerman et al., 2007). However, such comparisons between cancer patients receiving palliative care, patients with curative treatment intention (or mixed patient groups as typically found in hospitals of rehabilitation clinics), and the general population with sufficient sample sizes would be very helpful for clinicians in understanding the specific needs of patients with advanced cancer.

QoL is a multidimensional construct that is focused on the subjective assessment of the patients rather than objective criteria. One problem in QoL research is that the patients’ internal frames of reference they use for their QoL assessments can change over time as a result of adaptation processes. Such adaptation processes may help patients maintain a relatively high level of satisfaction; however, from a statistical point of view, it is problematic when the scales are evaluated with changing underlying frames of reference. The term “response shift” describes this phenomenon, and several methods have been developed to identify and quantify such response shift effects (Vanier Vanier et al., 2021; Schwartz et al., 2020; Ortega-Gómez et al., 2022). One approach is the use of anchoring vignettes (Topp et al., 2020; Grol-Prokopczyk, 2017; Au and Lorgelly, 2014; Crane et al., 2016; Salomon et al., 2004). These vignettes are descriptions of fictitious subjects, characterized by certain properties. When patients assess the persons described in these vignettes with regard to criteria such as health, it is possible to derive conclusions about the respondents’ frames of reference. Such anchoring vignettes have also been used in oncological studies (Korfage et al., 2007; Ilie et al., 2019). It has been shown that breast cancer patients (Hinz, 2017) and prostate cancer patients (Preiß et al., 2019) assessed such anchoring vignettes as being healthier than the assessments obtained from the general population, in other words, that such response shift effects did occur. To our knowledge, the technique of anchoring vignettes has not been applied yet to patients receiving palliative care. If it was found that the response shift effects that have already been demonstrated for cancer patients are even amplified in patients receiving palliative care, this would mean that the differences in QoL between these groups using standard questionnaires without taking response shift effects into account would actually underestimate the true differences. Therefore, in addition to the comparison of QoL assessments given by patients with and without palliative care intentions and the general population, we also intend to investigate such response shift effects.

In summary, the aims of this study were (a) to compare samples of cancer patients receiving palliative care, mixed cancer groups, and the general population regarding their QoL, and (b) to examine to what extent the assessments of anchoring vignettes can help better interpret these differences.

## Methods

### General procedure in selecting and matching the samples

In this study, we used a sample of cancer patients receiving palliative care, samples of mixed cancer patients, and samples of the general population. Since QoL assessments depend on sex and age, with stronger impairments in QoL for women and for older people (Nolte et al., 2019; Wedding et al., 2007), group comparisons are of limited value if the groups differ in sex and age distributions. To allow fair comparisons between groups, we chose the following approach. The numerically smallest group of palliative patients (*n* = 152) was retained in its entirety. From the other samples, both the samples of the mixed cancer patients and the samples of the general population, subsamples were selected in such a way that the sex and age distributions matched those of the palliative group. These matched subsamples then allowed bias-reduced comparisons between groups.

### Sample of cancer patients receiving palliative care

The study participants were consecutively recruited at the outpatient palliative-care clinic of the University Cancer Center Leipzig, Germany, between November 2020 and May 2022. Patients were eligible for the study if they had a confirmed cancer diagnosis and received palliative care. Exclusion criteria were insufficient command of the German language and severe cognitive impairment. A total of 152 (response rate: 61%) patients agreed to take part in the study and to complete the questionnaires. The mean age of this sample was 65.1 years, and 89 (58.6%) of the participants were females. The tumor sites with the highest frequencies were gastrointestinal tract (38.8%), female genital organs (21.1%), and breast (16.4%). Regarding therapy, 67.1% received surgery, 53.3% radiotherapy, 82.9% chemotherapy, and 24.5% hormone therapy. Further details of the sample are given elsewhere (Schnabel et al., 2023).

### Samples of mixed cancer patients

For the analysis of the EORTC QLQ-C30 scores, we used a sample that originally comprised 4,020 cancer patients who were consecutively recruited in five German study centers. We use the term ‘mixed cancer patients’ here to indicate that the patients had different types of cancer and that no distinction was made between palliative and curative treatment intentions. The mean age of that original sample was 58.4 years, and 2068 of the patients (51.4%) were females; for further details see (Mehnert et al., 2014). To form a sample that matched the group of palliative care patients in sex and age distribution, we selected a subsample from this sample with a mean age of 65.3 years and a proportion of women of 58.5%. This subsample consisted of 2,348 patients. The tumor sites with the highest frequencies were breast (24.3%), gastrointestinal tract (18.9%), and male genital organs (18.1%). Of these 2,348 patients, 74.1% received surgery, 46.8% radiotherapy, 48.1% chemotherapy, and 13.9% hormone therapy.

For the analysis of the assessments of the anchoring vignettes, we used a sample that originally comprised 1,108 consecutively recruited cancer patients treated in a German rehabilitation clinic. The mean age of the participants of that sample was 53.1 years, and 704 (63.5%) of them were females, see (Hinz et al., 2022). As with the sample of cancer patients who had completed the EORTC QLQ-C30, we again selected a subsample that matched well with the sex and age distribution of the palliative group. This resulted in a comparison group of 535 patients with a mean age of 65.1 years and a proportion of women of 56.3%. The most frequent cancer sites were breast (27.7%), gastrointestinal tract (20.7%), and prostate (22.2%). The proportions of therapeutic procedures were as follows: surgery (90.6%), radiotherapy (43.1%), chemotherapy (40.0%), and hormone therapy (20.6%).

### Samples of the general population

For the comparisons with the cancer patients, we used two general population samples, one for the comparison of the EORTC QLQ-C30 scores, and the other for the assessments of the vignettes. In both cases, the samples were fairly representative of the German general population in terms of age, gender, and education. Starting with more than 200 sampling points covering all regions of Germany, street, house, and flat were chosen with the random-route technique. Finally, the target person in the household was also selected randomly using the Kish-selection-grid technique.

The general population sample that served for comparisons in the EORTC QLQ-C30 was composed of two subsamples (*n* = 2,448 and *n* = 2028) of the German general population. In total, the sample of the general population comprised 4,476 subjects (Hinz et al., 2018). The mean age of the total sample was 49.8 years, and the percentage of women was 54.7%. After selecting a subsample to match sex and age distribution, the subsample comprised 2,141 persons with a mean age of 65.1 years and percentage of women of 54.7%.

The general population sample that served for comparisons of the anchoring vignettes originally consisted of 2,409 subjects (Hinz et al., 2016), the mean age of that original sample was 50.8 years, and 1,287 (51.1%) of them were women. After matching according to sex and age, a subsample of 1,229 persons remained, with a mean age of 65.1 years and a proportion of women of 58.6%.

All these studies with cancer patients and with samples of the general population were approved by the Ethics Committee of the Medical Faculty of the University of Leipzig, Germany, and written informed consent was obtained from all participants.

### Instruments

#### EORTC QLQ-C30

The QoL questionnaire EORTC QLQ-C30 (Aaronson et al., 1993) was designed to assess QoL in cancer patients. It comprises 30 items and includes five functioning scales (physical, role, cognitive, emotional, and social functioning), eight symptom scales, a scale concerning financial difficulties, and a global health/QoL scale. High scores (range: 0–100) in the functioning scales and in the global health/QoL scale indicate high levels of QoL, while high scores in the symptom scales mean low levels of QoL. In addition to these scales, we calculated an overall sum score (Giesinger et al., 2016) which is defined as the mean of the functioning scales and the (inverted) symptom scales.

### Anchoring vignettes

Self-rated health (SRH) was measured with the Visual Analogue Scale of the QoL questionnaire EQ-5D (Brooks, 1996). The participants were asked to assess their current state of health on a 0–100 scale, with the end points *worst possible health* (0) and *best imaginable health* (100). In addition, the participants were asked to assess two vignettes of patients regarding their health. These vignettes were presented as follows:

“Patient A is handicapped in his mobility due to a disease. He has problems using stairs, cannot perform his daily tasks (e.g., shopping), and occasionally has to use a wheel chair. He has hip and knee pain but considers it tolerable. Mentally he feels well. He is not anxious or depressed and does not see a reason to complain about his health.”

“Patient B has chronic back pain and physicians have been unable to figure out why. Although Patient B can move and fulfil his daily activities without help, he feels alienated by his pain, he mistrusts the physicians, and he perceives his future health situation as hopeless.”

These vignettes have already been used in studies with the general population (Hinz et al., 2016), with breast cancer survivors (Hinz, 2017), prostate cancer patients (Preiß et al., 2019), and patients suffering from cardiovascular diseases (Hinz et al., 2020).

### Statistical analysis

Mean score comparisons were expressed with Cohen’s effect sizes *d* that relate mean score differences to the pooled standard deviation of the groups. The statistical significance of group differences were first tested with one-way ANOVAs. In case of a significant result of this omnibus test, paired *t*-tests with Bonferroni correction were performed. There were three group comparisons for each variable. A significance of 0.05 is indicated if the calculated *p*-value falls below the value of 0.05/3 = 0.0167; the analogue threshold values for the significances of 0.01 and 0.001 are 0.0033 and 0.00033, respectively. All statistical analyses were performed with SPSS, version 27.

## Results

### QoL mean scores for the cancer groups and the general population

**Figure 1:**
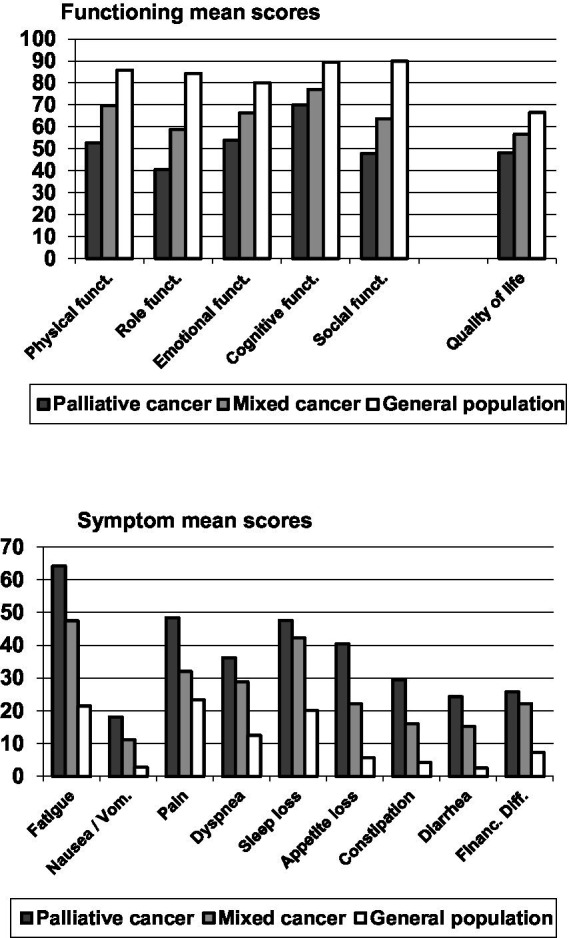
Mean scores of the scales of the EORTC QLQ-C30.

Figure 1 shows mean scores of the EORTC QLQ-C30 scales for the cancer patients receiving palliative care, the mixed cancer group, and the general population. On all functioning scales, the QoL of the palliative group was lowest, followed by the mixed cancer group. Best QoL was measured in the general population (see top graph of Figure 1). The same applied to the symptom scales, where high scores represent a high burden and, therefore, a low QoL.

A more detailed picture of these relationships is presented in the upper part of Table 1.

**Table 1:** Mean scores for palliative cancer patients, mixed cancer patients, and the general population.

Scale	Palliative cancer		Mixed cancer		Gen. popul.		Palliative cancer vs. Gen. popul.			Mixed cancer vs. Gen. popul.			Pallative cancer vs. Mixed cancer		
	*M*	SD	*M*	SD	*M*	SD	delta	*d*	*p*	delta	*d*	*p*	delta	*d*	*p*
EORTC QLQ-C30															
Physical	52.7	24.4	69.6	23.9	85.8	18.9	−33.1	−1.53	***	−16.2	−0.76	***	−16.9	−0.70	***
Role	40.5	30.1	58.8	34.9	84.3	24.7	−43.8	−1.60	***	−25.5	−0.86	***	−18.3	−0.56	***
Emotional	53.8	26.2	66.3	26.1	80.1	20.7	−26.3	−1.12	***	−13.8	−0.59	***	−12.5	−0.48	***
Cognitive	69.9	26.5	77.1	26.0	89.5	18.0	−19.6	−0.88	***	−12.4	−0.56	***	−7.2	−0.27	*
Social	47.9	32.4	63.7	33.0	89.9	20.4	−42.0	−1.59	***	−26.2	−0.98	***	−15.8	−0.48	***
Global QoL	48.2	19.1	56.6	22.9	66.5	20.6	−18.3	−0.92	***	−9.9	−0.46	***	−8.4	−0.40	***
Fatigue	64.2	24.2	47.5	30.5	21.4	23.8	42.8	1.78	***	26.1	0.96	***	16.7	0.61	***
Nausea/Vomiting	18.1	26.1	11.2	22.6	2.8	9.4	15.3	0.86	***	8.4	0.53	***	6.9	0.28	**
Pain	48.4	31.0	32.0	34.0	23.4	26.5	25.0	0.87	***	8.6	0.28	***	16.4	0.50	***
Dyspnoea	36.2	33.7	28.8	33.0	12.5	24.2	23.7	0.82	***	16.3	0.57	***	7.4	0.22	ns
Insomnia	47.6	33.6	42.3	37.4	20.2	28.5	27.4	0.88	***	22.1	0.67	***	5.3	0.15	ns
Appetite loss	40.4	35.9	22.2	33.5	5.7	15.8	34.7	1.34	***	16.5	0.67	***	18.2	0.52	***
Constipation	29.4	35.5	16.1	30.1	4.2	14.7	25.2	1.00	***	11.9	0.53	***	13.3	0.41	***
Diarrhea	24.3	33.1	15.3	28.8	2.5	10.9	21.8	0.99	***	12.8	0.64	***	9.0	0.29	**
Financial difficulties	25.8	32.9	22.2	32.3	7.3	19.4	18.5	0.71	***	14.9	0.58	***	3.6	0.11	ns
Sum Score	58.2	17.6	70.9	19.3	87.4	13.8	−29.2	−1.86	***	−16.5	−1.00	***	−12.7	−0.69	***
SRH and vignettes															
SRH	50.5	18.8	60.5	19.2	67.6	20.6	−17.1	−0.87	***	−7.1	−0.36	***	−10.0	−0.53	***
Vignette A	49.4	17.8	51.4	18.2	42.4	18.4	7.0	0.39	***	9.0	0.49	***	−2.0	−0.11	ns
Vignette B	41.1	18.5	41.2	19.0	44.6	17.2	−3.5	−0.20	ns	−3.4	−0.19	**	−0.1	−0.01	ns
Diff. SRH minus Vig. A	1.2	18.7	9.2	22.5	25.2	24.9	−24.0	−1.10	***	−16.0	−0.68	***	−8.0	−0.39	***
Diff. SRH minus Vig. B	9.6	24.5	19.4	25.5	22.9	25.9	−13.3	−0.53	***	−3.5	−0.14	ns	−9.8	−0.39	***

Delta: group mean difference; d: effect size; *p*, significance level; ^***^p < 0.001; ^**^p < 0.01; ^*^p < 0.05; ns, not significant; SRH, self-rated health.

With regard to the statistical significance of the overall group differences, the ANOVAs yielded a significance of 0.001 for all variables with one exception; for vignette B, the significance was 0.01. The significance levels for the pairwise group differences are given in Table 1. Regarding the comparison between the palliative group and the general population, the effect sizes are higher than the effect sizes of the comparisons with the mixed cancer group. Among the subscales, fatigue reached the highest effect size (*d* = 1.78), followed by role functioning (*d* = −1.60) and social functioning (*d* = −1.59). The comparison between the mixed cancer group and the general population also showed relatively high effect sizes for the subscales fatigue (*d* = 0.96), social functioning (*d* = −0.98), and role functioning (*d* = 0.86), while the differences in pain were relatively small (*d* = 0.28). Comparing the two groups of patients, the palliative group showed lower scores in all subscales, especially in physical functioning (*d* = −0.70), but also in fatigue (*d* = 0.61) and role functioning (*d* = −0.56), while the differences for insomnia (*d* = 0.15) and financial difficulties (*d* = 0.11) were relatively low.

In all three group comparisons, the effect sizes of the sum score (*d* between −0.69 and −1.86) were higher than those of the single scales, and in particular higher than the effect sizes of the 2-item global health/QoL scale (*d* between −0.40 and −0.92).

### Self-rated health (SRH) and assessment of the vignettes for the cancer groups and the general population

**Figure 2:**
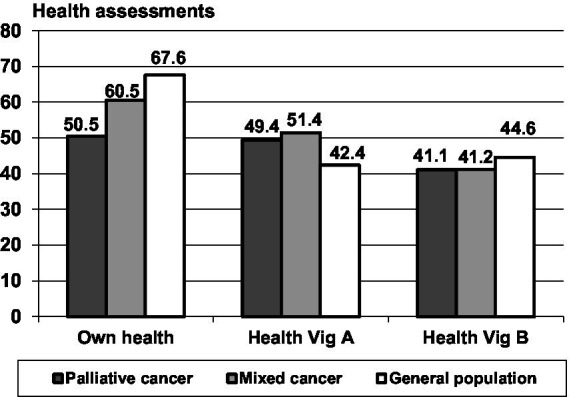
Self-rated health (SRH) and health ratings for vignettes A and B.

The SRH state (own health) was lowest in the group of palliative care patients and highest in the general population (Figure 2 and Table 1, lower part), a pattern that was also found for the EORTC QLQ-C30 sum score comparisons. Both patient groups rated Vignette A (predominantly physical health problems) as being significantly more healthy (*M* = 49.4 and *M* = 51.4) than the corresponding ratings obtained from the general population (*M* = 42.4), with only small differences between the patient groups. Concerning the assessments attributed to vignette B, this pattern did not occur; the patients assessed Vignette B as being even less healthy in comparison with the ratings obtained from the general population.

The relatively low scores for the respondents’ evaluation of their own health and the relatively high scores in the assessments of vignette A in the patients’ groups result in strong differences between the estimations of the own health and the health of Vignette A. Table 1, lower part, presents these relationships in more detail. While the participants of the general population rated their own health as being 25.2 points higher than that of vignette A, this difference was markedly lower in the cancer groups, only 9.2 points (mixed cancer group) and 1.2 points (palliative care group). The effect size for the comparison between the palliative care patients and the general population concerning this difference between SRH and ratings of Vignette A (*d* = −1.10) was even stronger than the difference in SRH alone (*d* = −0.87).

## Discussion

The first aim of this study was to compare the QoL scores of three groups: cancer patients receiving palliative care, mixed cancer patients, and the general population. As was to be expected, QoL was lowest in the group of patients receiving palliative care, and highest in the general population. This sequence was observed in all 15 dimensions of the EORTC QLQ-C30. This result underlines the importance of early, high-quality advance care planning, as the QoL of palliative patients is significantly impaired. A poorer QoL can, for example, be associated with a higher psychological burden, depression and even the patient’s wish to die (Elliesen et al., 2022). The highest effect sizes for the comparison between the palliative care group and the general population were observed for the sum score (*d* = −1.86) and for fatigue (*d* = 1.78). Regarding the comparison between the mixed cancer patients and the general population, fatigue showed the second highest effect size among the scales of the EORTC QLQ-C30 (*d* = 1.10). This supports previous findings that fatigue is a severe problem in cancer patients (Weis et al., 2017). In contrast to fatigue, pain showed a much lower effect size. This might be due to different approaches to responding to the pain-related items of the questionnaire. While patients may focus on cancer-related pain, members of the general population may also consider other kinds of pain, such as back pain or headache. Moreover, pain in the cancer groups might be treated with appropriate medication.

On two of the subscales, there were only relatively small differences between the two clinical groups: insomnia (*d* = 0.15) and financial difficulties (*d* = 0.11). This means that sleep problems and the perceived financial toxicity of the cancer disease in patients with curative treatment are nearly as intense as in the group of patients receiving palliative care, and should, therefore, be focused upon by health care providers since these dimensions generally do not receive much attention in QoL considerations.

The global health/QoL mean score of the patients receiving palliative care was slightly below the mean of the 0–100 range (*M* = 48.2) and similar to mean scores reported in other studies with cancer patients receiving palliative care, e.g., *M* = 42.1 (Ben-Arye et al., 2021), *M* = 45.2 (Kyranou and Nicolaou, 2021), *M* = 46.3 (Valero-Cantero et al., 2023), and *M* = 53 (Davda et al., 2021), but lower than the mean scores of a study from Lebanon (*M* = 65.8 (Chaar et al., 2018)) and a study focusing on four Nordic countries [*M* = 66.3 and *M* = 65.4 (Liposits et al., 2021)]. Several factors might have an impact on the way how patients assess their QoL: cultural differences, differences in the general healthcare systems, delayed diagnoses and treatment, barriers to care and costs of treatment. In addition, there are several models of delivery of palliative care (Hui, 2019; Zimmermann et al., 2023), which have an impact on the quality of palliative care. Interdisciplinary interventions led by palliative medicine specialists have generally resulted in more positive outcomes compared to interventions led solely by physicians (Hui, 2019).

The sum score of the EORTC QLQ-C30 proved to be a very useful compilation of the QoL facets. In all three group comparisons the effect sizes of this sum score were higher than most of the single scales. The 2-item global health/QoL scale was much less sensitive in this respect. Previous research with the EORTC QLQ-C30 has also shown that the differences on the 2-item global health/QoL scale are smaller than the aggregation of the single dimensions (Hinz et al., 2017). This can be explained by two factors. First, the aggregation of multiple components increases the statistical power. Second, it is possible that global assessments of health-related aspects are more prone to response shift effect than specific aspects (Hinz et al., 2017), as discussed below.

The EORTC QLQ-C30 proved to be an effective instrument for measuring QoL even in patients receiving palliative care, who did not appear overwhelmed by completing all 30 questions. Other studies with patients receiving palliative care similarly used this questionnaire successfully (van Roij et al., 2018). There is a specific questionnaire for cancer patients receiving palliative care, the EORTC QLQ-C15-PAL (Groenvold et al., 2006; Hansen et al., 2022). This shorter questionnaire with 15 items is easier for patients to complete than a 30-item instrument. However, the EORTC QLQ-C30 allows for a comparison of the results with those of other clinical groups and the general population. Until now, there have been several normative studies for the EORTC QLQ-C30, e.g., (Nolte et al., 2019; Nolte et al., 2020), which is not the case for the C15-PAL. Therefore, we recommend the use of the longer 30-item instrument even in the case of palliative care.

The second research question focused on whether the cancer patients and, in particular, the patients receiving palliative care, changed their frame of reference in evaluating their QoL. For that reason, we used the vignettes and tested the hypothesis that the patients would rate the health status of the subjects described in the vignettes as being higher than the general population did. Regarding vignette A with mainly physical problems, the hypothesis was clearly confirmed: the mean assessments reported by the patients were higher than the assessments of the general population, which means that the patients tended to tolerate more health problems and to consider a mediocre health status as rather acceptable in comparison with the judgments of the general population. In contrast to the expectations, however, the palliative care group did not rate vignette A as being healthier than the mixed cancer group did. This points to a response shift from the pre-cancer time to the time after cancer diagnosis (which can be inferred from the difference between the mixed cancer group and the general population). However, this effect does not seem to be amplified when the patients transit to the state of palliative care. One possible interpretation for the lack of response-shift differences between the two clinical groups could be that the changes in the frame of reference occur primarily when confronted with a potentially life-threatening disease, and that there is no longer a linear relationship between detriments in QoL and changes in the frames of reference. This is a surprising and new finding and requires elaboration in further studies. If confirmed, this finding would suggest that QoL assessments among cancer patients receiving palliative care may be broadly comparable to those of other cancer patients without major distortion from response shift. However, further research is needed to verify this across different domains and patient populations.

Vignette B with mainly mental health problems did not show such response shift effects. This suggests that changes in the frames of reference mainly occur in the area of physical health, a result that was also found in a previous study (Preiß et al., 2019). While the term QoL includes both, physical and mental components, response shift phenomena seem to only affect the physical components. However, the lack of response shift effects with Vignette B may also be due to the specific design of this vignette. Moreover, mental health conditions may be considered more stable or constant in comparison with physical health conditions.

When implementing early integration into palliative care (Lakhani et al., 2022) and choosing appropriate assessments of QoL, it is important to bear in mind that the real detriments can be underestimated due to response shift effects, at least regarding the physical domains of health. This also points to the need to repeatedly record symptoms, QoL, and supportive care needs of patients during the course of the disease.

Assessing the QoL of cancer patients as accurately as possible is important in order to be able to better evaluate the effect of therapeutic measures in cancer treatment and to prove the effect of interventions to improve QoL (Lee et al., 2025; Nguyen et al., 2023; Sebri and Pravettoni, 2023).

Some limitations of the study should be mentioned. Both clinical groups are not necessarily representative of the underlying populations of patients. In the palliative care group, the patients were recruited via the outpatient clinic, which might result in a lower proportion of patients in the final stage of the disease. In the mixed cancer group that was recruited in a rehabilitation clinic, both patients with low detriments as well as those with very strong detriments might be underrepresented. Regarding the differences between the two groups of cancer patients, one further limitation is that the groups also differ with respect to cancer type distribution and treatment to a certain degree. We did not perform sensitivity and power analyses on the matching between the groups, and the matching was based only on age and gender, although other factors such as social status or disease spectrum may have contributed to the group differences.

For comparisons of the QoL data with the general population, we used data from a general population study. In the meantime, other internet-based general population studies have been performed (Nolte et al., 2020) whose use might have resulted in slightly different group comparisons. Moreover, the EORTC QLQ-C30 was originally designed for cancer patients and not for the general populations which may introduce limitations such as floor effects or interpretational challenges.

The vignettes used in our study have their specific characteristics. Different vignettes with regard to age, sex, and complaints might also have resulted in other assessments.

Summing up, the results of this study present a detailed description of QoL of patients receiving palliative care with respect to two comparison samples: mixed cancer patients and the general population. The response shift effects underline that the differences obtained in studies that compare cancer patients and the general population tend to underestimate the actual differences.

## Data Availability

The raw data supporting the conclusions of this article will be made available by the authors, without undue reservation.
